# Surgical Treatment of Ovarian Pregnancy

**DOI:** 10.1155/2021/6618751

**Published:** 2021-02-24

**Authors:** Efthymia Thanasa, Ioannis Thanasas, Nikoleta Koutalia, Maria Mousia

**Affiliations:** ^1^Medical School, Aristotle University of Thessaloniki, Thessaloniki, Greece; ^2^Department of Obstetrics and Gynecology, General Hospital of Trikala, Trikala, Greece; ^3^Department of Pathology, General Hospital of Trikala, Trikala, Greece

## Abstract

The description of this case concerns the early diagnosis and the surgical treatment of a patient diagnosed with an ectopic ovarian pregnancy. A gravida 2, para 0 woman with a history of termination of pregnancy in the second trimester, was referred to the outpatients of the Gynecologic Department of the General Hospital of Trikala, reporting vaginal bleeding, accompanied by a deep, mild pain in the abdomen for a few days. The urine pregnancy test was positive. The transvaginal ultrasound in combination with the *β*-chorionic gonadotropin level was indicative of an ectopic pregnancy, and the surgical treatment of the patient was decided. Intraoperatively, the presence of an ovarian ectopic pregnancy was detected, and a wedge resection of the affected ovary was performed. The patient was discharged from our clinic on the third postoperative day, with instructions for weekly follow-up of the *β*-chorionic gonadotropin level until it returns to normal values.

## 1. Introduction

Ectopic pregnancy is defined as the pregnancy in which the fertilized ovum is implanted outside the endometrium of the normal uterine cavity [1]. The exact etiological mechanism behind the implantation and development of the ectopic blastocyst has not yet been fully elucidated. In general, a history of pelvic surgery, previous ectopic pregnancy, use of intrauterine contraceptive devices, infertility, and a history of pelvic inflammatory disease are factors associated with a high incidence of ectopic pregnancy [2, 3]. The ectopic pregnancy is estimated to affect the 1.2%-1.4% of all pregnancies, while in the 95% of cases, it is located within the fallopian tube [4]. Extratubal localizations of the ectopically located trophoblasts are reported in the ovary (our case), in the midline of the fallopian tube, the cesarean section scar, the cervix, and in the peritoneal cavity, accounting for about 5% of all ectopic pregnancies [5].

## 2. Case Report

**Figure 1:**
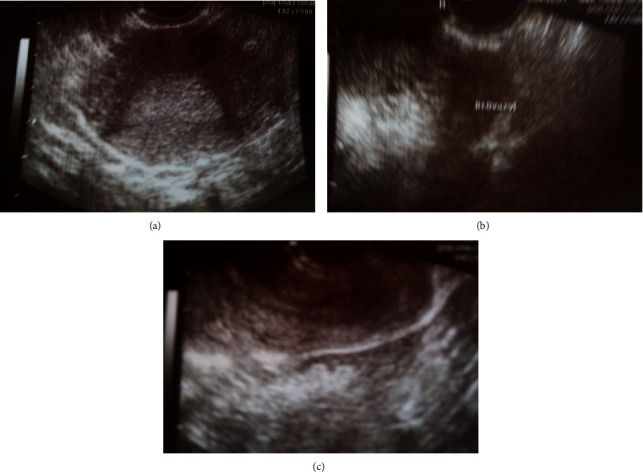
(a) Transvaginal ultrasound: the absence of a gestational sac in the endometrial cavity supports the diagnosis of an ectopic pregnancy (our case). (b) Transvaginal ultrasound: the presence of mass in the anatomical area of the adnexa supports the diagnosis of an ectopic pregnancy (our case). (c) Transvaginal ultrasound: the presence of blood clots in the pouch of Douglas supports the diagnosis of an ectopic pregnancy (our case).

The case concerns a gravida 2, para 0 woman, 27 years old with a history of termination of pregnancy in the second trimester, due to fetal chromosomal abnormalities, who was referred to the gynecologic outpatients due to vaginal bleeding, accompanied by a deep, mild pain in the lower abdomen for the last few days. The patient has been diagnosed with a pregnancy of undetermined location for about ten days. Based on her last menstrual period, her current pregnancy was calculated at 8 weeks and 2 days. The follow-up with a quantification of *β*-chorionic gonadotropin levels every second day revealed a nonreassuring development of the fetus (1st sample = 4113 mlU/mL, 2nd sample = 3904 mlU/mL, 3rd sample = 4207 mlU/mL), which in addition could be indicative of an ectopic pregnancy. The laboratory values at the time of admission were Ht 33.9%, Hb 10.8 gr/dl, PLT 243 × 103/ml, WBC 9.90 × 103/ml, and NEUT 79.9%, while the checkup of both the coagulation mechanism and biochemical control was without pathological findings. The bimanual gynecological examination revealed severe sensitivity during the movement of the cervix and the palpation of the right adnexa. The transvaginal ultrasound (Figures 1(a)–1(c)) indicated the absence of a gestational sac within the endometrial cavity and the presence of an inconclusive mass in the anatomical area of the right adnexa, as well as blood clots in the pouch of Douglas.

**Figure 2:**
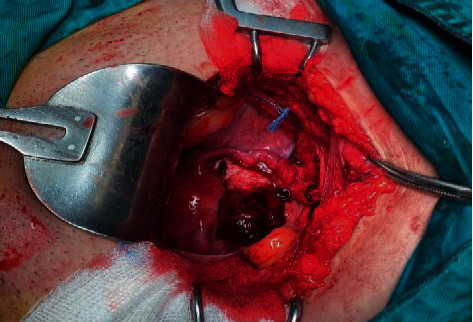
Intraoperative finding of an ectopic ovarian pregnancy (our case).

**Figure 3:**
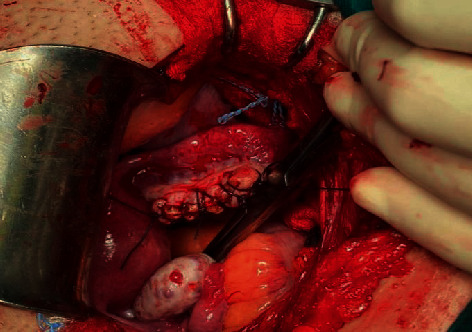
Surgical treatment of the ectopic ovarian pregnancy with wedge resection of the affected ovary and suturing of the ovarian tissue (our case).

**Figure 4:**
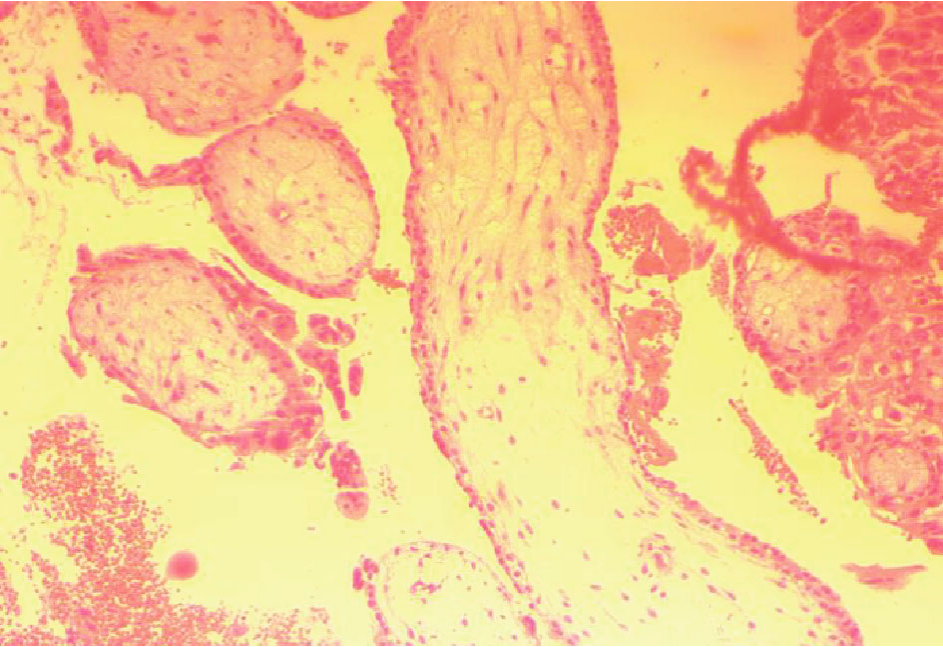
Histological picture of an ectopic ovarian pregnancy (our case).

The combination of the ultrasound findings with the quantification of *β-*HCG and the clinical status of the patient raised the diagnosis of an ectopic pregnancy, and the surgical treatment of the patient was decided, due to the estimated impending hemodynamic instability. Intraoperatively, a swelling of the right ovary was observed, with the presence of a bleeding reddish mass on its surface but without the participation of the corresponding fallopian tube in the lesion (Figure 2), while free blood and blood clots were apparent within the peritoneal cavity, as well as in the pelvis. The diagnosis of a possible ectopic ovarian pregnancy was made, and a wedge resection and suturing of the affected ovary was performed (Figure 3). The histological examination of the surgical specimen confirmed the diagnosis (Figure 4). After an uncomplicated postoperative course and declining *β*-HCG levels, the patient was discharged on the 3rd postoperative day. Three weeks later, the *β*-HCG level was zero.

## 3. Discussion

The ovarian ectopic pregnancy was first described by Mercureus in 1614 [6]. In this medical entity, the implantation of the fertilized ovum may involve the inner of the ovarian cortex (primary) or the surface of the ovary (secondary) [7]. The ovarian pregnancy is the most common form of a nontubal ectopic pregnancy and is estimated to account for about 0.5%-3% of all cases [8]. The primary ovarian pregnancy is rarer and is estimated to affect the 1/6000 to 1/40000 of all pregnancies [9]. Nevertheless, the increased vascularity that characterizes a pregnancy and the proximity of the ectopically implanted trophoblast with the ovarian and uterine vessels can lead to a massive and life-threatening bleeding [10].

The exact etiological mechanism of the ovarian ectopic pregnancy has not yet been fully elucidated. The main risk factor that has so far been proven responsible for the implantation of the fertilized ovum in the area of the ovary is the use of contraceptive devices. It is estimated that the risk of an ovarian ectopic pregnancy is much higher among women with an intrauterine contraceptive device compared to the general population, as well as in women undergoing in vitro fertilization processes with embryo transfer (IVF–ET). Other risk factors include a history of pelvic inflammatory disease and pelvic surgeries, which are highly associated with the incidence of tubal pregnancies as well [11]. Moreover, there are few cases of ectopic ovarian pregnancies in the international literature, which have been occurred after a subtotal hysterectomy [12]. More rarely, an ovarian pregnancy can occur without the presence of the classic previously described risk factors (our case) [13].

The preoperative diagnosis of an ovarian pregnancy is not easy [14]. The clinical manifestations do not differ substantially from an ectopic tubal pregnancy [15]. A history of secondary amenorrhea, abdominal pain of variable intensity, and abnormal vaginal bleeding are the main clinical features [16]. In the event of a ruptured ovarian pregnancy, the differential clinical diagnosis must include the rupture of a corpus luteum cyst or an ovarian cyst's torsion [13].

In addition to the clinical criteria, the contemporary use of the transvaginal ultrasonography in combination with the quantification of *β*-HCG levels has significantly increased the diagnostic accuracy of this medical entity. Ultrasound findings such as the absence of an intrauterine pregnancy and the presence of a gestational sac on the surface or inside the ovarian cortex support the diagnosis of an ovarian ectopic pregnancy. The transvaginal ultrasound plays a key role in the preoperative diagnosis of an unruptured ovarian pregnancy, while in cases of ruptured ones, there are no typical ultrasound findings that could differentiate it from a ruptured tubal pregnancy or a ruptured corpus luteum cyst [17]. Furthermore, the use of MRI nowadays can be useful in the diagnosis of an ovarian pregnancy, especially in cases where the ultrasound findings are ambiguous or not conclusive [18].

The diagnosis of the ovarian ectopic pregnancy is, in the majority of cases, being made intraoperatively, and it is confirmed by the histological examination of the surgical specimen (our case) [19]. Spielberg's (1878) diagnostic criteria, such as the noninvolvement of the corresponding fallopian tube in the lesion, the presence of a gestational sac in the ovary, the connection to the uterus through the ovarian ligament, and the presence of ovarian tissue in the wall of the sac, at multiple and different sites, can be too strict to confirm the diagnosis, resulting possibly into a significant underestimation of the prevalence of the disease [20]. As a result, nowadays, the noninvolvement of the fallopian tube and the simultaneous proven presence of chorionic villi within the ovaries consist the modified criteria based on which *σθππορτ* the diagnosis of ovarian pregnancies (our case) [21]. Finally, in those cases where the clinical and imaging findings are not conclusive and there is a severe diagnostic problem, laparoscopy may assist in the diagnosis, providing the advantage of the concomitant treatment of the disease as well [22].

The treatment of the ovarian ectopic pregnancy is divided into surgical and conservative, depending on the time of the initial diagnosis. Concerning the conservative one, methotrexate is the most widely used drug with the best therapeutic results. Methotrexate therapy can be used in early stage patients with hemodynamic stability [23]. The classic surgical approach of the disease with open or laparoscopic access is the wedge resection of the ovary and the suturing of the remaining ovarian tissue (our case). For the case where the diagnosis is made late and is accompanied by severe bleeding, an oophorectomy or adnexectomy may be required [24].

The prognosis depends mainly on the gestational age and the erosive activity of the ectopic trophoblast. Spontaneous bleeding caused after ovarian rupture is the main complication of the disease, with significantly increased rates of maternal morbidity and mortality. In cases of ovarian pregnancy after the early surgical treatment of the disease, the success rates for future pregnancies are considered to be very satisfactory [25].

## 4. Conclusion

The modern diagnostic approach of the ectopic ovarian pregnancy is a very important step towards the successful treatment of this rare but at the same time life-threatening obstetric complication. The early recognition of the symptoms and the risk factors associated with this medical entity, as well as the correct application of the modern and advanced technology allows nowadays the early diagnosis and the immediate treatment of the disease, in order to reduce the increased risk of maternal morbidity and mortality.

## Data Availability

The data that support the findings of this study are available from the corresponding author upon reasonable request.
